# Environmental enrichment alters protein expression as well as the proteomic response to cocaine in rat nucleus accumbens

**DOI:** 10.3389/fnbeh.2014.00246

**Published:** 2014-07-21

**Authors:** Cheryl F. Lichti, Xiuzhen Fan, Robert D. English, Yafang Zhang, Dingge Li, Fanping Kong, Mala Sinha, Clark R. Andersen, Heidi Spratt, Bruce A. Luxon, Thomas A. Green

**Affiliations:** ^1^Department of Pharmacology and Toxicology, The University of Texas Medical BranchGalveston, TX, USA; ^2^Center for Addiction Research, The University of Texas Medical BranchGalveston, TX, USA; ^3^Department of Biochemistry and Molecular Biology, The University of Texas Medical BranchGalveston, TX, USA; ^4^Sealy Center for Molecular Medicine, Institute for Translational Science, The University of Texas Medical BranchGalveston, TX, USA; ^5^Department of Preventative Medicine and Community Health, The University of Texas Medical BranchGalveston, TX, USA

**Keywords:** differential rearing, drug addiction, drug abuse, label-free quantification, proteomics, cocaine

## Abstract

Prior research demonstrated that environmental enrichment creates individual differences in behavior leading to a protective addiction phenotype in rats. Understanding the mechanisms underlying this phenotype will guide selection of targets for much-needed novel pharmacotherapeutics. The current study investigates differences in proteome expression in the nucleus accumbens of enriched and isolated rats and the proteomic response to cocaine self-administration using a liquid chromatography mass spectrometry (LCMS) technique to quantify 1917 proteins. Results of complementary Ingenuity Pathways Analyses (IPA) and gene set enrichment analyses (GSEA), both performed using protein quantitative data, demonstrate that cocaine increases vesicular transporters for dopamine and glutamate as well as increasing proteins in the RhoA pathway. Further, cocaine regulates proteins related to ERK, CREB and AKT signaling. Environmental enrichment altered expression of a large number of proteins implicated in a diverse number of neuronal functions (e.g., energy production, mRNA splicing, and ubiquitination), molecular cascades (e.g., protein kinases), psychiatric disorders (e.g., mood disorders), and neurodegenerative diseases (e.g., Huntington's and Alzheimer's diseases). Upregulation of energy metabolism components in EC rats was verified using RNA sequencing. Most of the biological functions and pathways listed above were also identified in the Cocaine X Enrichment interaction analysis, providing clear evidence that enriched and isolated rats respond quite differently to cocaine exposure. The overall impression of the current results is that enriched saline-administering rats have a unique proteomic complement compared to enriched cocaine-administering rats as well as saline and cocaine-taking isolated rats. These results identify possible mechanisms of the protective phenotype and provide fertile soil for developing novel pharmacotherapeutics. Proteomics data are available via ProteomeXchange with identifier PXD000990.

## Introduction

It is clear that humans display vast individual differences in susceptibility to drug addiction. Some people become addicted after a single exposure yet others can be resistant to addiction even after many exposures to high doses of drug. Understanding the mechanisms leading to resistance to addiction will provide new targets for the treatment or even the prevention of addiction. To identify novel targets, addiction science needs good animal models of resistance to addiction.

The environmental enrichment paradigm is an animal model of resistance to addiction. In this paradigm, rats are assigned either to an enriched condition (EC) with daily exposure to novelty (children's toys), exercise, and social contact with conspecifics (i.e., group housing), or an isolated condition (IC; single housed with no novelty). Our work and others' has shown that enrichment increases sensitivity to the locomotor activating and conditioned place preference effects of stimulants yet produces a decrease in intravenous self-administration of cocaine or amphetamine (Bowling and Bardo, 1994; Bardo et al., 1995; Green et al., 2002, 2010; Thiel et al., 2009, 2010). This decrease in self-administration has been described for acquisition, maintenance, extinction and reinstatement phases, using fixed and progressive ratios and in both prevention (i.e., before drug exposure) and treatment (during abstinence) models.

Currently there are no FDA approved pharmacotherapeutics for cocaine addiction in the United States despite decades of study of currently known targets. Thus, identifying completely novel targets for pharmacotherapeutic and genetic intervention is paramount to the success of developing a viable treatment for addiction. Accordingly, the first step in the process is determining intrinsic differences between the EC and IC brain and then examining how each group responds to cocaine. Our prior EC/IC cocaine research has focused on gene transcription and transcription factors (Green et al., 2010; Pavlovsky et al., 2013); the current project utilizes an unbiased discovery-based proteomic method looking for differential expression of *proteins* in the nucleus accumbens of EC and IC rats self-administering cocaine or saline with the goal of discovering proteins and/or biochemical pathways that could serve as targets in the treatment of cocaine addiction.

The analyses employed for this study exploit known functional relationships to identify over-representation of related protein groups in two complementary analyses. First, the Ingenuity Pathways Analysis (IPA) interrogates a curated database of published protein functional relationships (Kramer et al., 2014) to identify coordinated regulation of protein lists derived using a traditional *p*-value cutoff. Second, a Gene Set Enrichment Analysis (GSEA; in this case using proteins) uses a running-sum statistic of all rank-ordered proteins (i.e., no *p*-value threshold) (Subramanian et al., 2005).

## Materials and methods

### Subjects

Male Sprague-Dawley rats (40) arrived at 21 days of age and were randomly assigned to either an EC or an isolated condition to remain throughout the experiment. Enriched rats were housed 10 per cage in a large cage (77 × 78 × 60 cm) with 14 hard plastic children's toys replaced daily. Isolated rats were single housed in standard polycarbonate cages. Isolated rats were chosen as the control group rather than pair-housed rats because pair-housed rats represent an intermediate enrichment condition. Rats had ad lib access to food and water and were kept on a 12 h light/dark cycle. All experiments were conducted during the light phase of the cycle. All animal procedures were approved by the UTMB Institutional Animal Care and Use Committee and conform to the NIH Guide for the Care and Use of Laboratory Animals.

### Self-administration

To minimize differences in self-administration among EC and IC rats, all rats were first trained to bar-press for sucrose pellets (Noyes 45 mg). Prior to sucrose training (beginning PND51) rats were brought down to 85% of their free-feed body weight over 5 days. Rats were first trained on a continuous schedule of reinforcement in standard two-lever operant chambers (Med-Associates, St. Albans, VT); the schedule was incremented daily until the rats were on an FR5 schedule. All sessions lasted 15 min. After rats learned the FR5 schedule, they were allowed to regain their 100% free feed body weight prior to catheter surgery. For surgery, rats were anesthetized with ketamine (100 mg/kg IP) and xylazine (10 mg/kg IP) and a Silastic catheter (Fisher Scientific, Pittsburgh, PA) was fed into the jugular vein down to the heart. The catheter exited from the back from a stainless steel threaded pedestal. After 1 week of recovery, rats were placed back into the operant chamber and allowed to self-administer 0.5 mg/kg cocaine (National Institute on Drug Abuse, Bethesda, MD) or saline given in 0.1 ml over 5.8 s under a continuous schedule of reinforcement. Sessions lasted 2 h daily for 14 days, and the session terminated when the rat received 30 infusions to eliminate EC/IC differences in cocaine intake that could confound proteomic results. Control rats self-administered saline. Thus, the study was a 2 (EC/IC) X 2 (cocaine/saline) design. The number of sessions (14) was chosen because sensitization, tolerance and transcriptional changes are all stable at 14 days (Miller et al., 1998; Alibhai et al., 2007; Green et al., 2008). For the final session (PND88), nucleus accumbens tissue was harvested 3 h after the start of the self-administration session.

### Protein extraction

Nucleus accumbens (NAc) tissue samples from the right side of two rats in same group were combined for protein extraction, washed with ice cold tris-buffered saline (TBS), homogenized on ice in a buffer containing TBS pH 7.4, 1% Igepal-CA630 (NP-40), 1× protease inhibitor cocktail, 20 mM NaF, 1 mM Na_3_VO_4_, 10 mM DTT, and 5 mM EDTA, and then centrifuged at 750 g for 20 min at 4°C. The pellet, containing the nuclear fraction, was set aside. The top fraction was transferred to new tubes and centrifuged at 20,000 g for 20 min at 4°C. Methanol/chloroform (v/v 1:4:1) was added to the resulting supernatant, and it was kept at room temperature 15–30 min, vortexing every 5 min, and centrifuged at 16,000 g for 20 min at 4°C. Finally, 500 uL of acetone was added to remove the methanol and chloroform. The 20,000 g pellet fraction was treated similarly to remove lipids (membrane fraction). All fractions were then dissolved in a buffer containing 6 M urea, 1% NP-40, 20 mM Tris–HCl pH 7.4, 1× protease inhibitor cocktail, 10 mM DTT and 5 mM EDTA, and stored at −80°C until further analysis.

### Preparation of samples for LC-MS

Equal amount of protein extracts from each stock (100 μg) was digested with trypsin (sequence grade modified trypsin, Promega, trypsin:protein = 1:333 molar ratio), and incubated at 37°C overnight. The peptide mixture was extracted with a C18 tip (Pierce), and eluted with 90% acetonitrile in 0.1% TFA. The extracted peptides were dried and resuspended in 5 mM Tris, pH 7.4 (25 μL) for LC-MS analysis.

### Nanoflow liquid chromatography-mass spectrometry (nanoLC-MS/MS) analysis

Peptide mixtures for EC-control, IC-control, EC-cocaine and IC-cocaine samples were block randomized and analyzed by nanoLC-MS/MS using a nanoLC chromatography system (nanoLC 1D plus, Eksigent), coupled on-line to LTQ-Orbitrap Velos mass spectrometer (Thermo Fisher Scientific, San Jose, CA) through a nanospray ion source (Thermo Scientific). Chromatographic columns (75 μm × 10 cm) and trap columns (75 μm × 1 cm) were packed with 5 μm Zorbax SB-C18 (Agilent, Santa Clara, CA). After equilibrating in 95% solvent A (0.1% formic acid in water) and 5% solvent B [0.1% formic acid in acetonitrile (ACN)], the samples (7 μL in 5 mM Tris, pH 7.4) were injected onto the trap column and subsequently eluted (400 nL/min) by gradient elution as follows: isocratic at 5% B, 0–5 min; 5 to 35% B, 5–75 min; 35 to 95% B, 75–80 min; and isocratic at 95% B, 80–90 min. Total run time, including column equilibration, sample loading, and analysis was 104 min.

All LC-MS/MS data were acquired using XCalibur, version 2.1.0 (Thermo Fisher Scientific). For high-resolution data-driven analyses (DDA), the survey scans (*m/z* 300–2000) (MS) were acquired in the Orbitrap at 60,000 resolution (at *m*/z = 400) in profile mode, followed by six CID fragmentation MS/MS spectra, acquired in centroid mode. The automatic gain control targets for the Orbitrap were 5 × 10^5^ for the MS scans and 1 × 10^4^ for MS/MS scans. The maximum injection times for the MS1 and MS/MS scans were 300 and 500 ms, respectively. For MS/MS acquisition, the following settings were used: parent threshold = 50,000; isolation width = 2.0 Da; normalized collision energy = 35%; activation time = 30 ms. Monoisotopic precursor selection, charge state screening, and charge state rejection were enabled, with rejection of singly charged and unassigned charge states. Dynamic exclusion was used to remove selected precursor ions (±10 ppm) for 60 s after MS/MS acquisition. A repeat count of 1 and a maximum exclusion list size of 500 was used. The following ion source parameters were used: capillary temperature 275°C, source voltage 2.0 kV, source current 100 uA, and S-lens RF level 63%.

### Proteomic data analysis

MS files (.raw) were imported into Progenesis LC-MS (version 4.1, Nonlinear Dynamics) for *m/z* and retention time alignment. The top 5 spectra for each feature were exported (charge deconvolution, top 1000 peaks) as a combined.mgf file for database searching in PEAKS (Ma et al., 2003) (version 6, Bioinformatics Solutions Inc., Waterloo, ON) against a combined UniProtKB/SwissProt rat-mouse database (September 2013 version, 20,264 proteins), appended with the common Repository of Adventitious Proteins (cRAP) contaminant database (The Global Proteome Machine, http://www.thegpm.org/cRAP/index.html). PEAKS DB (Zhang et al., 2012) and Mascot (version 2.3.02, Matrix Science) searches were performed with a parent ion tolerance of 20 ppm, fragment ion tolerance of 0.8 Da, and variable modifications of oxidation (M) and phosphorylation (STY). Trypsin was specified as the enzyme, allowing for 2 missed cleavages and a maximum of 3 PTMs per peptide. An additional search for unexpected modifications was performed with the entire Unimod database (Zhang et al., 2012). Finally, homology searching was performed using the SPIDER algorithm (Han et al., 2005) to identify peptides resulting from non-specific cleavages or amino acid substitutions. Mascot and PEAKS SPIDER searches were combined (inChorus), using a 1% false discovery rate cutoff for both search engines. The resulting peptide-spectrum matches (95% peptide probability) were imported into Progenesis LC-MS. Conflict resolution was performed manually to ensure that a single peptide sequence was assigned to each feature by removing lower scoring peptides. The resulting normalized peptide intensity data were exported, and the peptide list was filtered to remove non-unique peptides and all modified peptides except methionine oxidation. For quantification, the filtered list of peptide intensities was imported into Dante-R (Polpitiya et al., 2008; Karpievitch et al., 2009) (version 0.1.1), log_2_ transformed, and combined to protein abundances (RRollup) using the default settings, including one-hit wonders (50% minimum presence of at least one peptide, minimum dataset presence 3, *p*-value cutoff of 0.05 for Grubbs' test, minimum of 5 peptides for Grubbs' test). The resulting proteins were quantified by Two-Way ANOVA, and *p*-value adjustment was performed according to the Benjamini and Hochberg method (Benjamini and Hochberg, 1995).

### Gene set enrichment analysis (GSEA) analyses of protein

The IPA analyses involve deciding on a *P*-value cutoff for deciding if proteins are significantly differentially expressed. Highly stringent cutoffs introduce Type II error (false negatives) and less stringent cutoffs introduce Type I errors (false positives). To mitigate this problem, normalized protein intensities were analyzed via GSEA using the Broad Institute's GSEA software (http://www.broadinstitute.org/gsea). Protein names were converted to gene names by IPA. The advantage of the GSEA is that all proteins are ranked (not just the statistically significant) and significance is determined using a running-sum statistic for the whole gene set (Subramanian et al., 2005). Analyses included proteins from all 3 fractions. Gene sets were taken from the Broad Institute's (v.4.0) set of curated gene sets [C2.all; including Biocarta, Kyoto Encyclopedia of Genes and Genomes (KEGG) and Reactome], Gene Ontology gene sets (C5.all) and transcription factor target genes (C3.tft). Analyses were run with 1000 permutations of gene sets (size 15–500) using the Signal2noise ranking metric.

### RNA sequencing

The left nucleus accumbens from each rat was purified using the RNeasy kit using the manufacturer's directions. cDNA libraries were created by reverse transcribing the RNA and creating the second strand. Blunt ends were phosphorylated and “a-tailed” so that adapters could be ligated to both ends. RNA was sequenced with a HiSeq 1000 system from Illumina. Four samples were added to each lane. cDNA was amplified using “bridge” amplification. Base calls were made using fluorescently labeled nucleotides. Over 100 million reads were taken at 50 bp (paired-end reads). FastQC (v0.9.1) (Andrews, 2014) was used to check the quality of the reads. Reads were mapped to the rat reference genome (RN4) using Tophat2 (v2.0.4) (Kim et al., 2013) and Bowtie2 (v2.0.0.6) (Langmead and Salzberg, 2012) software packages. The R package EdgeR (v.3.0.8) (Anders and Huber, 2010; Robinson et al., 2010) was then used for analysis using the “trimmed mean for *M*-values” (TMM) method for normalization and tag-wise dispersion using “count” data. A likelihood ratio *F*-test was used for generating *P*-values to compare EC vs. IC rats. Transcripts significantly regulated (*p* < 0.05) were overlaid onto the IPA energy metabolism pathway for comparison to protein values.

## Results

The sessions were limited to 30 infusions to eliminate differences in self-administration among groups. The two rats with the slowest acquisition from each group were dropped from the experiment, leaving eight rats in each group. The remaining cocaine rats all acquired self-administration within 3 days and all rats received the maximum 30 infusions for each of the last five sessions (data not shown). There was also no EC/IC difference in time to complete 30 infusions. In several instances, data acquisition problems in the LC-MS/MS runs prevented proper alignment of samples for label-free quantification. In those cases, three samples per condition (corresponding to protein from six mice) were used for quantification and statistical analysis.

Table 1 contains a selection of significantly regulated proteins for the cocaine main effect, environmental enrichment main effect and interaction. A complete list of all identified proteins can be found in Supplementary Materials. Overall, 52 proteins were significantly regulated (*p* < 0.05; fold change >1.5) as a function of cocaine self-administration, with 11 proteins decreased and 41 proteins increased. In the case of environmental enrichment, 117 proteins were significantly regulated, with 50 decreased and 67 increased. The interaction between environmental enrichment and cocaine produced 128 significant proteins. For both the cocaine and environmental enrichment main effects, the protein exhibiting the greatest decrease was cysteine sulfinic acid decarboxylase (CSAD). Moreover, this protein had the largest positive effect size of the significant proteins in the Enrichment X Cocaine interaction term.

**Table 1:** **A selection of significantly changed proteins (*p* < 0.05; fold change > 1.5) for cocaine main effect, environmental enrichment main effect and cocaine X enrichment interaction**.

**Accession**	**Gene symbol**	**EC log2-fold**	**EC est fold**	**EC *p*-value**	**Coc log2-fold**	**Coc est fold**	**Coc *p*-value**	**Interaction log2 effect size**	**Interaction effect size**	**Interaction *p*-value**	**Fraction**
P61751	ARF4	−2.21	−4.63	0.013	−1.39	−2.61	0.022	2.60	6.06	0.016	S1
Q1WIM2	CADM2							−0.61	−1.53	0.033	S1
Q5I0K3	CLYBL				2.02	4.05	0.036				S1
Q64611	CSAD	−5.30	−39.50	0.020	−4.41	−21.27	0.031	7.21	148.14	0.021	S1
P24268	CTSD	1.32	2.49	0.035	1.15	2.22	0.047	−1.59	−3.00	0.050	S1
Q9JHL4	DBNL				−0.33	−1.25	0.020	0.42	1.34	0.023	S1
Q641Y8	DDX1	−0.62	−1.54	0.020				0.89	1.86	0.020	S1
Q63622	DLG2	1.62	3.08	0.033	1.60	3.03	0.033	−2.17	−4.49	0.036	S1
Q6PDL0	DYNC1LI2	1.50	2.82	0.043	1.50	2.82	0.043	−2.09	−4.25	0.044	S1
Q8BGY2	EIF5A2	1.10	2.15	0.045				−1.37	−2.59	0.047	P1
O54921	EXOC2	1.77	3.41	0.033	1.67	3.18	0.036	−2.27	−4.82	0.040	S1
P28741	KIF3A	2.23	4.68	0.043	2.25	4.76	0.042				S1
P17046	LAMP2				3.40	10.56	0.041				S1
P21396	MAOA	0.82	1.76	0.047							P1
P36506	MAP2K2	−2.25	−4.74	0.018				2.65	6.26	0.022	S1
P10637	MAPT	1.22	2.33	0.045							P1
Q69ZK9	NLGN2							−0.93	−1.90	0.048	S1
Q6PEC4	SKP1							0.91	1.88	0.032	S1
Q9JI12	SLC17A6				4.58	23.87	0.033				S1
Q62634	SLC17A7				4.55	23.43	0.031				S1
Q8BRU6	SLC18A2				0.93	1.91	0.044				S1
Q80ZA5	SLC4A10	3.02	8.10	0.021	3.63	12.37	0.018	−3.99	−15.90	0.024	S1
P07895	SOD2	0.91	1.88	0.049							S1
Q9CQN6	TMEM14C				2.97	7.84	0.036				S1
Q60930	VDAC2	0.68	1.61	0.030							P1

A complete list of proteins can be found in Supplementary Materials.

### Cocaine main effect

#### Confirmatory evidence

**Figure 1:**
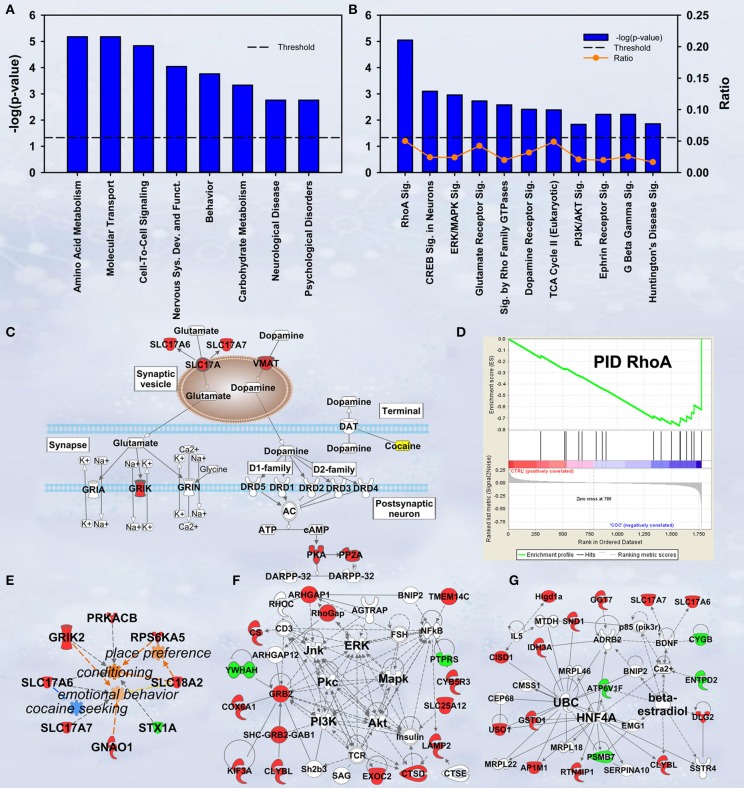
**IPA and GSEA findings for cocaine main effect**. A complete list of results can be found in Supplementary Materials, as can a complete list of all proteins in each pathway. **(A)** A selection of significant biological functions and diseases for cocaine main effect as determined by IPA. The y axis represents the −log(*p*-value) for each function/disease. **(B)** A selection of significant canonical pathways for cocaine main effect, as determined by IPA. Orange line represents a ratio of regulated proteins to all proteins in the pathway. **(C)** Combined glutamate and dopamine signaling pathways. Proteins whose expression levels were significantly increased are highlighted in red. SLC17A: vesicular glutamate transporter. GRIK: glutamate receptor (kainate). **(D)** GSEA plot demonstrating enrichment of proteins associated with the Protein Interaction Database's ras homolog family member A (RhoA) pathway. Protein regulation is ranked from those with highest correlation of expression to saline rats to the left to those with the highest correlation of expression in cocaine rats to the right. Vertical black tick marks represent relative rank of proteins in the RhoA pathway. **(E)** Significantly regulated proteins associated with cocaine-related behaviors. Proteins significantly increased are highlighted in red, while those that are significantly decreased are highlighted in green. **(F)** Network of regulated proteins related to kinase cascades. Solid lines represent direct interactions and dashed lines represent indirect. JNK: cJun N-terminal kinase. ERK: extracellular signal-regulated kinase. PKC: Protein kinase C. MAPK: mitogen-activated protein kinase. PI3K: phosphoinositide-3-kinase. AKT: v-AKT murine thymoma viral oncogene. **(G)** Network of regulated proteins related to ubiquitin C (UBC), hepatocyte nuclear factor 4alpha (HNF4A) and estradiol. Proteins significantly increased are highlighted in red, while those significantly decreased are highlighted in green. As a whole, this figure highlights previously-known effects of cocaine (dopamine signaling, RhoA signaling, glutamate signaling) and known kinase pathways (ERK, JNK, AKT, etc.) as well as novel findings (HNF4A, ubiquitination, etc.).

In discovery-based science it is important to have independent (i.e., orthogonal) validation of a subset of the results to provide greater confidence in the experiment as a whole. Luckily, the effect of cocaine on protein expression has been studied extensively. As a result, the published literature provides many validating measures. For cocaine main effect, the top-ranked biological functions (Figure 1A) were *Amino Acid Metabolism* and *Molecular Transport* [−log(*p*) = 5.18 for each]. Amino acid metabolic pathways lead to production of the neurotransmitters glutamate and dopamine, known to be important in cocaine addiction (see Figure 1C). Another highly ranked function, *Cell-to-Cell Signaling and Interaction* [−log(*p*) = 4.84], represents another important facet of the effects of cocaine on the brain and is also manifested in the significant canonical pathways as determined by IPA (Figure 1B). The top-ranked canonical pathway in IPA was *RhoA Signaling* [−log(*p*) = 5.05; Figure 1B], a pathway which has been demonstrated to show decreased activity in the NAcc by treatment with cocaine (Kim et al., 2009; Gourley et al., 2011). This result was also supported by the GSEA protein analysis showing significant regulation of the RhoA gene set curated by the Protein Interaction Database [Normalized Enrichment Score (NES) = −1.8, *p* < 0.005; Figure 1D; Table 2]. The 4th- and 7th-ranked canonical pathways were *CREB Signaling in Neurons* [−log(*p*) = 3.1] and *ERK/MAPK Signaling* [−log(*p*) = 2.9], respectively (Figure 1B; also see network in Figure 1F). These two signaling pathways are well known addiction-related signaling pathways (Berhow et al., 1996; Green et al., 2006; Larson et al., 2011). Further, the significant biological function named *Behavior* was comprised of *Conditioning* (*p* < 0.001), *Emotional behavior* (*p* < 0.005), *Cocaine-seeking behavior* (*p* < 0.01) and *Place preference* (*p* < 0.05) (see Figure 1E). Lastly, BDNF was identified as a significant upstream regulator (*p* < 0.01), confirming what is known about this protein in cocaine self-administration (Graham et al., 2007).

**Table 2:** **A selection of cocaine-regulated protein sets from the GSEA analysis**.

	**MSigDB collection**	**Proteins**	**Enrichment score**	**NES**	***p*-value**
**DOWNREGULATED BY COCAINE**					
MITOCHONDRIAL_ENVELOPE	C5	51	0.61	2.08	0.001
REACTOME_TCA_CYCLE_AND_RESPIRATORY ELECTRON_TRANSPORT	C2	82	0.53	1.91	0.001
KEGG_PARKINSONS_DISEASE	C2	65	0.51	1.77	0.001
KEGG_ALZHEIMERS_DISEASE	C2	68	0.49	1.77	0.001
REACTOME_RESPIRATORY_ELECTRON_TRANSPORT	C2	44	0.48	1.59	0.006
KEGG_HUNTINGTONS_DISEASE	C2	75	0.43	1.57	0.001
ANTI_APOPTOSIS	C5	21	0.56	1.55	0.034
**UPREGULATED BY COCAINE**					
PID_ER_NONGENOMIC_PATHWAY	C2	19	−0.79	−1.94	0.001
ST_INTEGRIN_SIGNALING_PATHWAY	C2	20	−0.78	−1.93	0.005
PID_RHOA_PATHWAY	C2	17	−0.77	−1.82	0.003
PROTEIN_SERINE_THREONINE_KINASE_ACTIVITY	C5	25	−0.69	−1.78	0.005
KEGG_WNT_SIGNALING_PATHWAY	C2	28	−0.67	−1.74	0.005
SIG_REGULATION_OF_THE_ACTIN_CYTOSKELETON BY_RHO_GTPASES	C2	17	−0.73	−1.67	0.012
KEGG_CHEMOKINE_SIGNALING_PATHWAY	C2	28	−0.64	−1.64	0.010
REACTOME_SEMAPHORIN_INTERACTIONS	C2	25	−0.62	−1.60	0.021

A complete list of proteins can be found in Supplementary Materials.

#### Glutamate/dopamine release

Our results show a significant increase for several proteins involved in glutamate and dopamine signaling—notably, we have significant increases in vesicular glutamate transporters (VGLUTs) 1 and 2 (SLC17A7 and SLC17A6, respectively) as well as the vesicular monoamine transporter VMAT2 (SLC18A2; Figure 1C).

#### Neurodegenerative proteins

Hungtingtin (HTT; *p* = 1.4E-5), microtubule-associated protein tau (MAPT; *p* = 3.8E-4), presenilin 1 (PSEN1; *p* = 0.003) and amyloid precursor protein (APP; *p* = 0.006) ranked 1st, 2nd, 7th, and 11th as protein upstream regulators, respectively. The GSEA protein analysis (see Table 2) revealed cocaine-induced decreases in KEGG pathways for Parkinson's (NES = 1.77, *p* < 0.001), Alzheimer's (NES = 1.77, *p* < 0.001) and Huntington's (NES = 1.57, *p* < 0.001).

#### Protein ubiquitination

Ubiquitin C (UBC) is a major hub in the network shown in Figure 1G. Note that expression for 6 of the 8 ubiquitin target proteins was increased by cocaine.

### Environmental enrichment main effect

#### Confirmatory evidence

**Figure 2:**
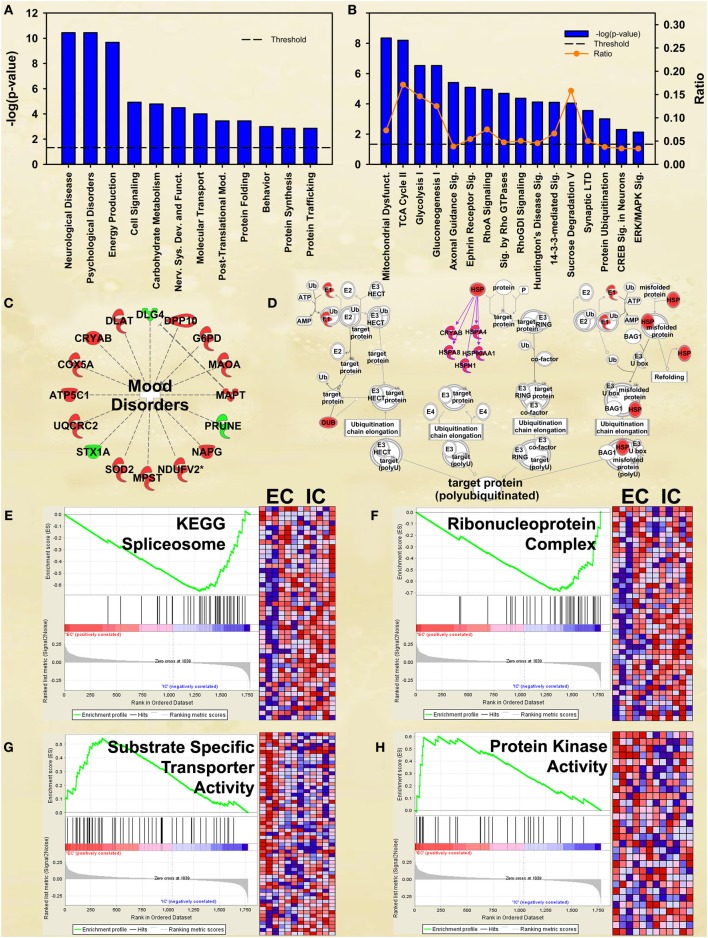
**IPA and GSEA findings for environmental enrichment main effect**. A complete list of results can be found in Supplementary Materials, as can a complete list of all proteins in each pathway. **(A)** A selection of significant biological functions and diseases for environmental enrichment main effect, as determined by IPA. The y axis represents the −log(*p*) value for each function/disease. **(B)** A selection of significant canonical pathways for environmental enrichment main effect, as determined by IPA. Orange line represents a ratio of regulated proteins to all proteins in the pathway. **(C)** Significantly regulated proteins associated with mood disorders as a subset of psychological disorders in Panel A. Proteins with a measured increase in expression level are highlighted in red, while those with a measured decrease in expression level are highlighted in green. **(D)** Protein ubiquitination canonical pathway. Proteins highlighted in red showed a measured increase in expression level. Symbols with purple outline are members of the heat-shock family of proteins (HSPs). DUB: de-ubiquitinating enzyme. **(E)** GSEA analysis of the KEGG *Spliceosome* gene set, showing a decreased expression of proteins relating to splicing in EC rats. Vertical black tick marks denote proteins in the gene set among ranked expression list of proteins. Heat map displays relative expression level of proteins in the set. Red denotes higher expression and blue denotes lower. **(F)** GSEA analysis using *Ribonucleoprotein complex* GO gene set, showing a decreased expression of proteins relating to splicing in EC rats. **(G)** GSEA analysis using GO *Substrate-specific transporter activity* gene set, showing increased expression of proteins related to transporter activity in EC rats. **(H)** GSEA analysis using GO *Protein kinase activity* gene set, showing upregulation in EC rats. This figure highlights the breadth of regulation of different novel pathways and protein sets regulated by enrichment.

Although not at the very top of the list of regulated pathways, the enrichment main effect IPA analysis also produced significant canonical pathway regulation of *CREB signaling in neurons* [−log(*p*) = 2.3] and *ERK/MAPK signaling* [−log(*p*) = 2.1; Figure 2B], supporting our own prior research (Green et al., 2010; Fan et al., 2013a,b). Also of interest is the regulation of multiple proteins associated with mood disorders (Figure 2C) as a subset of *Psychological disorders* (Figure 2A). In addition, the energy metabolism results described below confirm and greatly extend upon our earlier work (Fan et al., 2013a,b).

#### Ubiquitination and heat-shock proteins

EC rats had increased expression of several proteins in the *Protein ubiquitination* canonical pathway [−log(*p*) = 3.0; Figure 2D], particularly heat-shock proteins. These results agree with our prior research showing increases in heat-shock proteins in EC rats (Fan et al., 2013a).

#### Splicing and mRNA processing

EC rats exhibit decreased expression of proteins related to splicing, as measured by the GSEA protein analysis using the KEGG *Spliceosome* gene set (NES = −2.13, *p* < 0.001; Figure 2E; Table 3). This effect was driven, to a large degree, by U2-interacting small nuclear ribonucleoproteins and splicing-factor 3 proteins. In addition to the KEGG spliceosome gene set, the largely overlapping Gene Ontology (GO) gene set for *Ribonucleoprotein complex* was similarly regulated (NES = −2.28, *p* < 0.001; Figure 2F). In fact, the top seven scoring GO gene sets downregulated in EC rats were all related to splicing (NESs range from −2.28 to −1.59, *p*-values all less than 0.006).

**Table 3:** **A selection of environmental enrichment-regulated protein sets from the GSEA analysis**.

	**MSigDB collection**	**Proteins**	**Enrichment score**	**NES**	***p*-value**
**DOWNREGULATED IN EC**					
RIBONUCLEOPROTEIN_COMPLEX	C5	42	−0.69	−2.28	0.001
KEGG_SPLICEOSOME	C2	45	−0.65	−2.14	0.001
PID_MTOR_4PATHWAY	C2	22	−0.66	−1.86	0.003
KOYAMA_SEMA3B_TARGETS_UP	C2	20	−0.62	−1.71	0.005
TRANSMEMBRANE_RECEPTOR_ACTIVITY	C5	16	−0.59	−1.53	0.056
**UPREGULATED IN EC**					
REACTOME_TCA_CYCLE_AND_RESPIRATORY ELECTRON_TRANSPORT	C2	82	0.57	1.96	0.001
KEGG_PARKINSONS_DISEASE	C2	65	0.56	1.84	0.001
KEGG_HUNTINGTONS_DISEASE	C2	75	0.53	1.78	0.001
KEGG_ALZHEIMERS_DISEASE	C2	68	0.54	1.77	0.001
SUBSTRATE_SPECIFIC_TRANSPORTER_ACTIVITY	C5	57	0.54	1.74	0.001
KEGG_GLYCOLYSIS_GLUCONEOGENESIS	C2	28	0.63	1.73	0.002
ACTIVE_TRANSMEMBRANE_TRANSPORTER_ACTIVITY	C5	25	0.64	1.72	0.003
PROTEIN_KINASE_CASCADE	C5	30	0.60	1.69	0.003
REACTOME_SIGNALING_BY_RHO_GTPASES	C2	20	0.62	1.66	0.010
NEGATIVE_REGULATION_OF_APOPTOSIS	C5	29	0.58	1.62	0.012
STEIN_ESRRA_TARGETS_UP	C2	107	0.45	1.60	0.001
PHOSPHOPROTEIN_PHOSPHATASE_ACTIVITY	C5	18	0.62	1.54	0.026
NEUROGENESIS	C5	23	0.56	1.52	0.025

A complete list of proteins can be found in Supplementary Materials.

#### Transporters

One gene set robustly upregulated in EC rats was the GO gene set *Substrate-specific transporter activity* (NES = 1.74, *p* < 0.001; Figure 2G; Table 3). Proteins contributing to this effect were largely solute carriers (SLCs) and subunits of the Na+/K+ exchange pump. In contrast, calcium channels (CACNs) tended to be downregulated in EC rats.

#### Kinase activity

Another gene set robustly upregulated in EC rats is the GO gene set *Protein kinase cascade* (NES = 1.68, *p* < 0.005; Figure 2H; Table 3). Two of the top three proteins in the set (MINK1 and DBNL) are involved in receptor trafficking and endocytosis.

#### Energy metabolism

**Figure 3:**
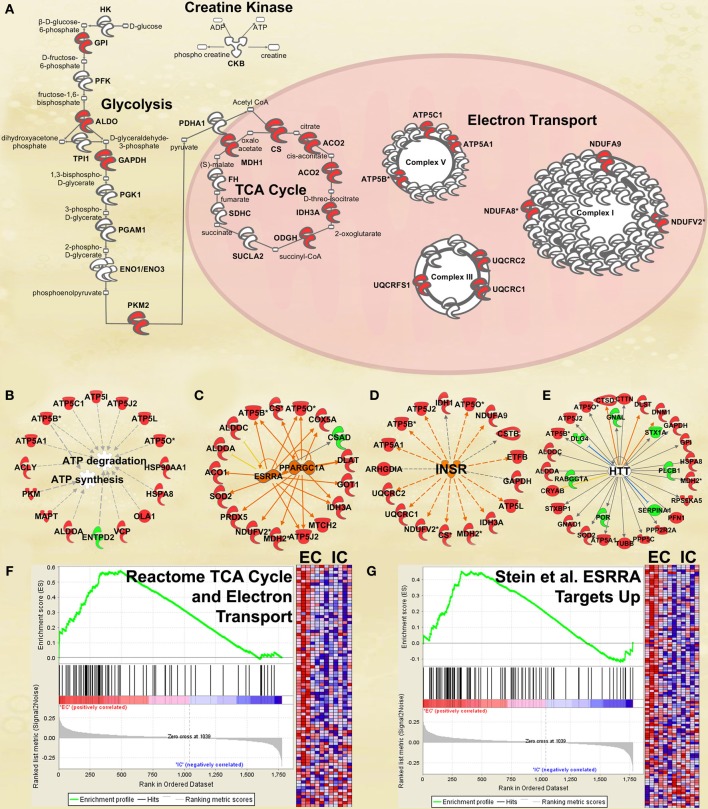
**IPA and GSEA findings illustrating the regulation of energy metabolism proteins by environmental enrichment**. A complete list of results can be found in Supplementary Materials, as can a complete list of all proteins in each pathway. **(A)** Canonical pathways involved in energy metabolism from IPA. Proteins whose expression was significantly increased in EC rats are highlighted in red. TCA: tricarboxylic acid. Complexes I–V: mitochondrial respiratory chain complexes. **(B)** Proteins involved in ATP synthesis and degradation, a subset from the IPA biological functions analysis of *Energy production* (Figure 2A). Proteins whose expression is increased are shown in red, while those with decreased expression are shown in green. **(C–E)** Proteins from Upstream Analysis in IPA whose expression supports upregulation of **(C)** PPARgamma coactivator protein 1alpha (PPARGC1A) and estrogen-related receptor alpha (ESRRA) activity, **(D)** insulin receptor (INSR), and **(E)** huntingtin (HTT). **(F)** GSEA analysis using Reactome *TCA Cycle and Electron Transport* gene set. **(G)** GSEA analysis using proteins upregulated by ESRRA from Stein et al. (2008). This figure focuses specifically on the depth of regulation of energy metabolism proteins by environmental enrichment.

One of the most regulated biological functions in EC vs. IC rats was *Energy production* (*p* = 2.12E-10; Figure 2A). Likewise, 4 of the top 5 canonical pathways were related to energy production (see Figure 2B), including *Mitochondrial dysfunction* [−log(*p*) = 8.3], *TCA cycle II* [−log(*p*) = 8.2], *Glycolysis I* [−log(*p*) = 6.5], and *Gluconeogenesis I* [−log(*p*) = 6.5]. Figure 3A depicts the consistent upregulation of these proteins in EC rats (particularly EC control rats) for the 4 canonical pathways. In addition, the significantly-regulated biological function *Energy production* was driven to a large degree by proteins related to ATP synthesis and degradation (Figure 3B). In support of the IPA results, the gene set with the highest normalized gene set enrichment score (NES) in the GSEA protein analysis was the Reactome *TCA cycle and respiratory electron transport* gene set (NES = 1.96, *p* < 0.001; Figure 3F; Table 3). The IPA upstream regulator analysis identified PPARGC1A as being activated by environmental enrichment (activation Z-score = 3.26, *p* = 6.8E-7). In addition, ESRRA (activation Z-score = 2.21, *p* = 2.96E-08) was also identified as an upstream regulator, with similar energy metabolism proteins in both results (Figure 3C). A subsequent literature search revealed that PPARGC1A interacts with ESRRA to increase expression of energy metabolism proteins (Ichida et al., 2002; Luo et al., 2003), offering good evidence for this mechanism driving the robust increases in energy metabolism proteins shown in Figure 3A. In support of the ESRRA results, a GSEA protein analysis identified a significantly upregulated gene set of proteins induced by ESRRA in a paper by Stein et al. (2008) (Figure 3G). It is important to note that the Stein et al. data are not part of the Ingenuity Knowledgebase, so the complementary IPA and GSEA results are completely orthogonal. In addition to PPARGC1A and ESRRA, the insulin receptor (INSR) was identified as a significant upstream modulator, with a clear majority of these proteins being related to energy metabolism (activation Z-score = 3.57, *p* = 9.4E-8; Figure 3D). The INSR, when activated, increases glucose uptake into cells (Weinstein et al., 2009) and signals through the PI3K/and ERK signaling cascades, both of which were identified by IPA as significant hubs [−log(*p*) = 1.8 and 2.13, respectively]. It is likely through transcriptional effects of these two mechanisms that INSR also increases expression of energy metabolism proteins.

#### Neurodegenerative proteins

Environmental enrichment has been shown to exert clearly beneficial effects on memory and cognition and to be beneficial in mouse models of Huntington's and Alzheimer's diseases (Hockly et al., 2002; Maesako et al., 2012). Among the proteins regulated by enrichment was the Alzheimer's-related protein MAPT (2.3 fold increase, *p* < 0.05). Ranking third in the biological function analysis in IPA was *Neurological disease* (Figure 2A), which included Huntington's disease (*p* = 6.1E-8) and Parkinson's disease (*p* = 2.8E-3). *Huntington's disease signaling* also appeared as a significant canonical pathway [−log(*p*) = 4.1; Figure 2B]. The top 3 upstream regulators identified by IPA were the Alzheimer's-related MAPT (*p* = 2.2E-25), APP (*p* = 4.1E-22), and PSEN1 (*p* = 9.2E-20) proteins. The HTT protein ranked an impressive 4th of all protein upstream regulators (*p* = 1.0E-10; Figure 3E) but showed only a trend for increased expression (1.4 fold increase, *p* = 0.08). GSEA confirmed increases in proteins related to KEGG pathways for Parkinson's (NES = 1.84, *p* < 0.001), Huntington's (NES = 1.78, *p* < 0.001) and Alzheimer's (NES = 1.77, *p* < 0.001) diseases.

### Enrichment by cocaine interaction

The interaction of enrichment X cocaine is potentially important because it shows how EC and IC rats react differently to cocaine.

#### Neurodegeneration

The construct *Neurological disease* was the top disease/biological function in IPA. This group included *Huntington's disease* (*p* = 3.8E-5). Again, MAPT (*p* = 4.1E-9), PSEN1 (*p* = 7.8E-8), HTT (*p* = 2.7E-7), and APP (*p* = 1.9E-6) were the top 4 upstream regulators.

#### Rho signaling

**Figure 4:**
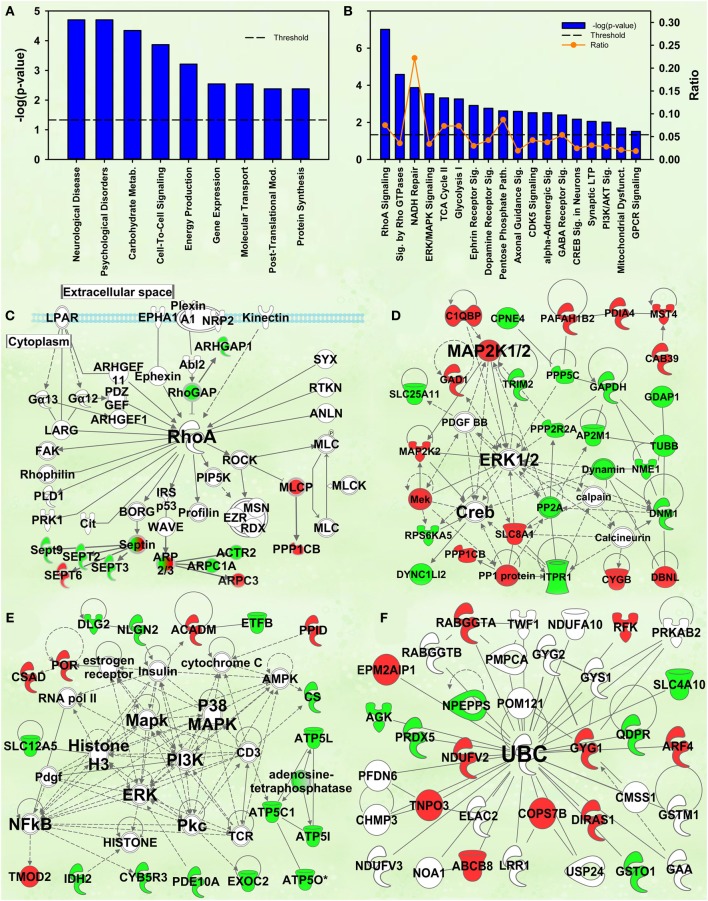
**IPA and GSEA findings for the Enrichment X Cocaine interaction**. A complete list of results can be found in Supplementary Materials, as can a complete list of all proteins in each pathway. **(A)** A selection of significant biological functions and diseases, as determined by IPA. The y axis represents the −log(*p*) value for each function/disease. **(B)** A selection of significant canonical pathways for Enrichment X Cocaine interaction, as determined by IPA. Orange line represents a ratio of regulated proteins to all proteins in the pathway. **(C)** *RhoA signaling* canonical pathway from IPA (from **B**). Proteins with a measured positive effect size are highlighted in red, while those with a measured negative effect size are highlighted in green. **(D)** Protein interaction network highlighting ERK, MAPK kinase 1/2 (MAP2K1/2) and cAMP response element binding protein (CREB) signaling. **(E)** Protein interaction network highlighting PI3K, MAPK, ERK and nuclear factor kappa B (NFkB) as hubs. **(F)** Protein interaction network demonstrating changes in expression of ubiquitin targets. The interaction term is important because it specifically identifies targets and pathways differentially regulated by cocaine in EC and IC rats.

The second- and third-ranking canonical pathways were *RhoA signaling* [−log(*p*) = 7.0] and *Signaling by Rho family GTPases* [−log(*p*) = 4.6], respectively (Figure 4B). Inspection of protein intensities revealed that IC control rats have less Rho-related proteins than the other groups and that IC rats showed induction after cocaine (Figure 4C).

#### Kinase signaling cascades

Several protein interaction networks were identified for the interaction term, including those in Figures 4D,E scoring 52 and 33, respectively. Both of these networks have hubs of known kinases (ERK, PI3K, p38, etc.) and downstream transcription factors (CREB, NFKB) all known to be important in the response to cocaine (Berhow et al., 1996; Russo et al., 2009; Larson et al., 2011). Further, our prior research has shown some of these to be important in environmental enrichment (Green et al., 2010; Fan et al., 2013a,b). The interpretation of these results is that EC and IC rats show differential kinase responses to cocaine, a result intriguing given that EC rats have a protective addiction phenotype.

#### Ubiquitination

Prior research from this laboratory demonstrated that EC and IC rats had differential expression of ubiquitin target proteins and these rats exhibited differential regulation of ubiquitin targets after restraint stress. The IPA analysis of the current data also suggests ubiquitination may be important for the differential expression of proteins in EC and IC rats after cocaine. Two different high-scoring networks (scoring 29 and 24) were identified with UBC as the major hub (the higher-scoring network is shown in Figure 4F).

#### Energy metabolism

Closer inspection of the heat maps from Figures 3F,G reveals that the EC main effect for energy metabolism proteins is mostly driven by EC saline-administering rats (left 3 columns). Not surprisingly, the IPA analysis for the interaction of enrichment and cocaine produced a significant result for *Energy production* (*p* = 6.14E-4; Figure 4A). For canonical pathways, *TCA cycle II* [−log(*p*) = 3.3] and *Glycolysis I* [−log(*p*) = 3.2] ranked 11th and 12th among regulated pathways, respectively (Figure 4B). When the GSEA data are reanalyzed comparing the EC saline vs. all other groups, 20 gene sets related to energy production and mitochondria were statistically significant, all with NES scores greater than 1.75 and *p*-values < 0.005. Thus, EC control rats had drastically higher levels of energy metabolism proteins than all other groups. In addition, a set of genes comprising genes with the ESRRA response element in the promoters also produced a significant result (NES = 1.49, *p* < 0.005). It is notable that this gene set is completely orthogonal to the Stein et al. and the IPA knowledgebase, meaning that 3 independent analyses suggest EC saline rats have higher expression of ESRRA target proteins.

### Confirmation of energy metabolism upregulation in EC rats *via* RNA-seq

**Figure 5:**
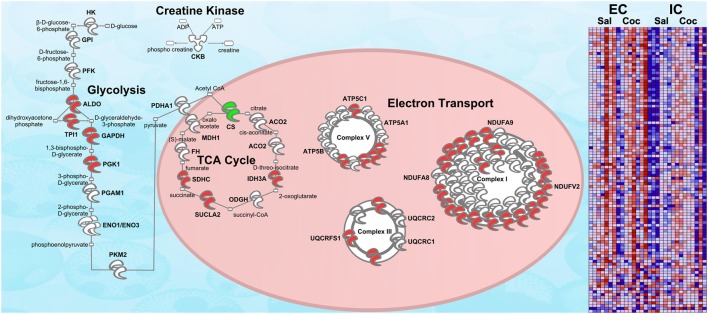
**RNA-seq upregulation of energy metabolism gene expression**. Transcripts whose expression was significantly increased in EC rats (main effect) are highlighted in red. Green denotes a decrease in expression. *N* = 7–8. This figure is an RNA validation of enrichment-induced upregulation of energy metabolism proteins (compare to Figure 3A). A complete list of all proteins in this pathway can be found in Supplementary Materials. Right: heat map of relative expression intensity of energy metabolism transcripts. Red denotes higher expression and blue denotes lower.

Figure 5 shows the same IPA pathway as Figure 3A, only this time overlaid with environmental enrichment main effect RNA-seq data. As with the protein data, one of the top-regulated canonical pathways was *Mitochondrial dysfunction* [−log(*p*) = 9.22]. Further, ESRRA was identified as a significant (*p* < 0.05) upstream regulator with an activation Z-score of 2.29, nearly identical to the protein data (*Z* = 2.21).

## Discussion

The results of this experiment provide compelling evidence that EC and IC rats have significant detectable differences in protein expression in the nucleus accumbens and that EC and IC rats show differential protein regulation after 14 days of cocaine self-administration. Our confidence in the novel EC/IC results is extremely high because the cocaine analysis provided more than a dozen results confirming what is already known about cocaine exposure. Further, in most cases the GSEA results support the IPA results even though the data sets comprising the IPA and GSEA functional databases are often completely orthogonal.

The most clear and striking difference between EC and IC rats is in the expression of proteins involved in energy production. Increased energy demand might be expected given that the brains of EC rats are more active than IC rats, but the clear Enrichment X Cocaine interaction for these proteins suggests that EC rats respond to cocaine differently than IC rats. In looking at the heat maps it is clear that EC control rats have the highest amounts of energy-producing proteins and that cocaine self-administration decreases these proteins to levels similar to those of IC rats. IC rats, by contrast had lower levels already and no further decrease after cocaine. Energy metabolism proteins were also differentially regulated by psychological stress in EC and IC rats in our prior experiments (Fan et al., 2013a,b). Thus, it is becoming increasingly clear that EC and IC brains respond differently to perturbations that affect energy metabolism. The protein results are backed by RNA-seq data confirming upregulation of the energy metabolism pathway at the mRNA and protein level. Thus, protein and mRNA both suggest the energy metabolism pathway as a whole is upregulated in EC rats.

Given that energy metabolism proteins are expressed at extremely high levels and have a rapid turnover (a result of the oxidative stress of the mitochondria), one might expect a better correlation between individual protein and mRNA expression. The lack of an exact match between protein and mRNA illustrates the main weakness of –omics studies: high Type II error (i.e., false negatives). Regardless, the analysis strategy employed in this study is designed to be somewhat resistant to both Type I and Type II error. The fact that the separate protein and mRNA analyses both identified *Mitochondrial Dysfunction* as a major factor attests to the resilience of the analysis. The fact that IPA and GSEA concur despite differential statistical strategies and functional annotation databases adds further confidence to the overall analysis strategy.

One recurring theme in this study as well as our upcoming transcriptomics study using mRNA from these same rats and our previous EC/IC stress proteomic study (Fan et al., 2013a,b) is proteins involved in neurodegeneration (Alzheimer's, Parkinson's, and Huntington's diseases). In the current study, MAPT was upregulated in EC rats, and MAPT, PSEN1, APP and HTT were among the most significant upstream regulators in multiple analyses. In trying to make sense of the results, it is interesting to note that mitochondrial dysfunction and energy metabolism are key players in the pathology of these neurodegenerative diseases (Quintanilla et al., 2013; Wang et al., 2013; Camilleri and Vassallo, 2014). Given that the mitochondrial proteins of EC rats are upregulated, one might hypothesize that environmental enrichment might alter the course of these neurodegenerative diseases. Published literature confirms that enrichment is protective in animal models of all three neurodegenerative diseases (Hu et al., 2010; Wood et al., 2010).

Much study has focused on the separate roles of VMAT2 and VGLUTs in addiction, but recent research suggests glutamate and dopamine are co-released in the NAcc of mice by mesolimbic dopaminergic neurons (Tecuapetla et al., 2010). Although it has not been shown that these neurotransmitters are packaged into the same vesicles, the current results showed concordant increases of VMAT2, VGLUT1, and VGLUT2 protein levels after cocaine. A literature search reveals that knockout of either VMAT2 or VGLUT2 can increase sensitivity to cocaine (Wang et al., 1997; Alsio et al., 2011). It has been shown previously that short-term cocaine exposure increases VMAT2 (Schwartz et al., 2007) yet long-term cocaine users have decreased VMAT2 (Little et al., 2003). Intriguing opportunities for regulation arise when one imagines fast ionotropic glutamate neurotransmission overlaid with slower metabotropic dopaminergic neuromodulation. Thus, it is possible that the coordinated increases in expression in the current study are relevant to the addictive nature of cocaine. Increased expression of these vesicular transporters might potentiate dopamine and glutamate release in the NAcc. Indeed, it has been shown that repeated cocaine administration potentiates glutamate release in addition to potentiating dopamine release (Reid and Berger, 1996). The authors propose that this pathway represents a fast mechanism for reward signaling.

For both main effects and the interaction, the largest effect size of regulated protein was cysteine sulfinic acid decarboxylase (CSAD). CSAD is the rate-limiting enzyme in the biosynthesis of taurine, an antioxidant and agonist of glycine receptors (Wang et al., 2005). Acute cocaine exposure has been shown to decrease taurine tissue concentration in the rat NAcc at 2 h post injection (Li et al., 2012). Additionally, repeated cocaine was shown to increase *extracellular* release of taurine at 30 min post injection (Yablonsky-Alter et al., 2009). The significance of these findings is unclear at this point, but the evidence at hand suggests taurine may play some role in addiction.

Taking a step back to get a broad view of the results, this study further supports the idea that the neurobiology of enriched rats differs significantly from that of isolated rats, particularly expression of proteins related to energy metabolism, RNA processing, membrane transporters and kinases. Further, EC rats show fundamentally distinct responses to cocaine compared to IC rats, particularly proteins related to RhoA signaling, energy metabolism, a variety of kinase cascades and ubiquitination. Interestingly, the EC-saline group was most distinct from the other three groups. Current and future experiments will begin to uncover the importance of these proteins in mediating this protective phenotype in an attempt to illuminate why many people are resistant to addiction despite exposure to drugs of abuse, thus identifying new targets for treatment of those who do become addicted. Future experiments teasing apart individual aspects of the enrichment condition (e.g., novelty, social contact, and exercise alone) will shed additional light on the exact nature of environmental enrichment.

## Author contributions

Cheryl F. Lichti assisted in experimental design for proteomics experiments, performed proteomics data analysis and assisted in writing of manuscript. Xiuzhen Fan performed experiments and assisted in experimental design. Robert D. English assisted in proteomics experimental design, performed LC-MS analysis and assisted in writing materials and methods. Yafang Zhang and Dingge Li were involved with behavioral, surgical, dissection and design aspects of the project. Fanping Kong, Heidi Spratt, Mala Sinha and Bruce A. Luxon were involved with design and execution of the primary and secondary RNA-seq analyses. Thomas A. Green participated in all aspects of experimental design, performed GSEA analysis, generated relevant figures, and participated in writing of the manuscript. All authors read, provided feedback and approved the final version of the paper.

### Conflict of interest statement

The authors declare that the research was conducted in the absence of any commercial or financial relationships that could be construed as a potential conflict of interest.
